# A role for Dps ferritin activity in long-term survival of *Escherichia coli*

**DOI:** 10.1128/spectrum.01837-24

**Published:** 2025-09-02

**Authors:** Zlatas Serebnitskiy, Katie Orban, Steven E. Finkel

**Affiliations:** 1 Department of Biological Sciences, Molecular and Computational Biology Section, University of Southern California 5116, Los Angeles, California, USA; University of Florida College of Dentistry, Gainesville, Florida, USA

**Keywords:** stationary phase, ferritin, Dps, survival

## Abstract

*Escherichia coli* expresses three ferritins that store acquired iron by oxidizing soluble Fe(II) to insoluble Fe(III), which can accumulate and later be utilized in cellular processes. Although bacterioferritin (Bfr) and ferritin (FtnA) sequester more Fe(III) atoms per multimeric complex, the abundance of the DNA-binding protein from starved cells (Dps), coupled with its preference for hydrogen peroxide as an oxidant in its ferroxidase activity, makes it a fundamental component in iron homeostasis and long-term stationary phase (LTSP) survival. To investigate the temporal role and mechanisms of action of Dps in parallel with the other ferritins, growth yield, survival, competitive fitness, and siderophore assays were performed under different conditions of iron availability. *dps* mutant strains exhibit the lowest cell viability and long-term cellular survival rates under all conditions tested, including iron toxicity, iron starvation, and in standard growth medium, in both competition and monoculture. We postulate that Dps is the critical ferritin, since under both monoculture and co-inoculation conditions*, ftnA* and *bfr* mutants maintain higher population densities during LTSP, while *dps* mutant viability decreases by up to four orders of magnitude. Furthermore, the production of siderophores, indicative of cells sensing iron starvation, is ~2,000 times greater in a *dps* mutant compared to the wild-type, *ftnA,* or *bfr* mutant strains, indicating that cells lacking Dps cannot appropriately maintain iron homeostasis.

**IMPORTANCE:**

Iron is a crucial cofactor for many enzymatic processes; however, it can be toxic due to its role in Fenton chemistry and production of reactive oxygen species (ROS). *E. coli* encodes three differentially expressed iron detoxifying and storage proteins, the ferritins FtnA, Bfr, and Dps, that contribute to maintaining iron homeostasis. While the distinct roles for each ferritin remain poorly elucidated, Dps is postulated to account for the majority of iron storage in stationary phase and later due to its abundance in stationary phase cells. Furthermore, its use of hydrogen peroxide as its preferred oxidant is hypothesized to help modulate the deleterious effects of intracellular iron and ROS production. Given that virtually all bacteria encode at least one *dps* homolog and most microbes in nature frequently experience a state akin to long-term stationary phase, the data presented here support a model in which Dps plays a fundamental role in long-term bacterial iron homeostasis and survival.

## INTRODUCTION

Iron plays an essential role in biological systems as a cofactor, catalyst, and electron carrier in all three domains of life (1). While essential, iron poses a significant risk to cells due to its role generating oxidative stress agents through two non-enzymatic Fenton reactions (2, 3):


(1) $$\begin{array}{rl} Fe^{2+}+H_{2}O_{2} & \to [FeO{]}^{2+}+H_{2}O\\ {}[FeO{]}^{2+}+H^{+} & \to Fe^{3+}+HO\cdot \end{array}$$

(2) $${\text{Fe}}^{3\;+}+{\text{H}}_{2}^{\;}{\text{O}}_{2}^{\;}⟶{\text{Fe}}^{2\;+}+{\text{H}}^{+}+\text{HOO}\cdot$$

Under physiological conditions, iron can take the soluble form of Fe(II), a reactive substrate in Fenton chemistry, or Fe(III) which is insoluble and less prone to produce free-radicals. Ferritins are highly conserved multimeric 24-mer iron detoxifying and storage complexes, observed to combat the production of reactive oxygen species (ROS), while helping to maintain iron concentrations necessary for cellular functions and iron homeostasis (4, 5). The ferroxidase centers in ferritins use molecular oxygen to oxidize Fe(II) to Fe(III), which is then stored and hypothesized to be released from the ferritin core, as necessary (6, 7).

In addition to the two canonical ferritins, ferritin (FtnA) and bacterioferritin (Bfr), most bacteria encode a third ferritin initially identified for its DNA-binding capacity, the DNA-binding protein from starved cells (Dps) (8). While functionally similar, little sequence homology exists between the canonical ferritins compared to Dps (8). Furthermore, distinct structural and enzymatic differences have been observed (9). FtnA and Bfr function as ~500 kDa 24-mers that store up to 4500 Fe(III) atoms per complex (4–6, 9). Bfr also contain 12 heme moieties, interfaced between two subunits that are suggested to play a role in the release of iron reserves (9–11). Comparatively smaller, Dps stores ~500 Fe(III) atoms as an ~250 kDa 12-mer (8, 12). Despite the lack of sequence homology, the three-dimensional structure of the 4 α-helices comprising the core of each subunit monomer is virtually identical (8). Despite the structural similarities of the subunits, another significant difference between FtnA and Bfr compared to Dps is the number and location of ferroxidase centers within each multimer. While FtnA and Bfr each have 24 ferroxidase centers, one for each monomer, Dps complexes contain 12 ferroxidase centers, shared between 2 adjacent subunits (13–16). In these ferroxidase centers, Dps primarily utilizes hydrogen peroxide as an oxidizing agent without generating hydroperoxyl or hydroxyl radicals via intrinsic catalase activity (1), enhancing iron detoxification and oxidative stress alleviation (12). Furthermore, Dps in *E. coli*, and many other species, is unique among ferritins due to its dual function as a DNA-binding protein (17). During the stationary phase, Dps is the most abundant nucleoid-associated protein in the cell (18) and is responsible for organizing the chromosome into a tightly packed, three-dimensional nucleoprotein lattice structure referred to as the biocrystal (19). Dps-DNA biocrystal formation has been demonstrated to protect cells from extremes of pH, heat, and both UV and gamma irradiation (20, 21); some studies also suggest a role for Dps in global gene regulation (18, 22, 23).

In many environmental niches, iron is often found to be a growth-limiting resource for bacteria (24, 25). In response to these limitations, bacteria have developed sophisticated iron acquisition systems, frequently using high affinity (*K*_b_ > 10^30^ [26]) iron-chelating ligands known as siderophores (1, 27, 28). Under conditions of low iron availability, bacteria synthesize siderophores and secrete them into the environment to bind and sequester extracellular ferric iron for subsequent intracellular uptake (29). Fe(III)-siderophore complexes require dedicated receptors that allow for energy-dependent transport into the cell (28). After transport through the outer and inner membranes, the reducing environment of the cytoplasm converts Fe(III) to Fe(II), solubilizing the iron; this can include further enzymatic processes that cleave the siderophore (27).

Consideration of population dynamics is essential when examining iron metabolism in the context of long-term survival. In laboratory batch culture, *E. coli* undergoes five distinct phases: lag phase, logarithmic or exponential phase, stationary phase, death phase, and long-term stationary phase (LTSP) (30, 31). Following inoculation into a fresh culture, bacteria undergo a lag phase, where the population is adapting to the influx of additional nutrients and prepares for vegetative growth. Next, cells transition to the logarithmic or exponential phase, characterized by rapid population growth, frequently with a generation time for *E. coli* of ~20 min in a rich medium such as Luria-Bertani Broth (LB). As environmental conditions change, the population density peaks and cells enter stationary phase where growth is arrested, but the population can remain at high density for 1–2 days in LB medium, depending on the strain and specific culture conditions (32–34). Eventually, the death phase occurs with an ~99% decrease in viable cell counts plateauing as cells transition into LTSP, where a batch culture population can survive for months or years without the addition of nutrients at relatively high cell densities (30–33, 35–37).

Using strains that lack each of the ferritins, we measured survival of the wild-type and each mutant strain in monoculture and under competitive conditions. We also measured siderophore production under different conditions of iron availability. Our data indicate each mutant exhibits differing growth and survival patterns, as well as different iron homeostasis phenotypes, suggesting each ferritin plays a role during specific phases of the bacterial lifecycle. Furthermore, we propose that Dps plays the most critical role of the three ferritins as *dps* mutants are significantly less fit than the wild-type strain or mutants that lack either of the two canonical ferritins. This is likely due to Dps’ role as both a significant iron sink and iron reservoir, depending on environmental and intracellular iron levels, coupled with its ability to lower ROS concentrations and neutralizing the potential deleterious effects of hydrogen peroxide during long-term stationary phase.

## MATERIALS AND METHODS

### Bacterial strains and ferritin mutant construction

All strains used in this study are a derivative of ZK126 (Δ*lacU169 tna-2*), a W3110-lineage *Escherichia coli* K-12 strain (34). The *dps* (SF2080, kanR), *ftnA* (SF2433, strR), *bfr* (SF2426, nalR), *ftnA dps::kan* (SF2609*,* kanR), *bfr dps::kan (SF*2610*,* kanR), *ftnA bfr (SF*2611), and *ftnA bfr dps::kan (SF*2612*,* kanR), mutant strains were constructed via P1 transduction using knockout mutants from the KEIO collection (38), allowing for viable cell counts of all strains in co-inoculations experiments (39). For competition assays, isogenic wild-type strains ZK1142 or ZK1143 are marked with nalidixic acid- or streptomycin-resistance, respectively (34, 36).

### Culture conditions, media, and titering assays

Overnight cultures were inoculated from frozen 20% glycerol stocks stored at −80°C. All cultures were inoculated into 5 mL LB Broth (Lennox) (10 g/L tryptone, 5 g/L yeast extract, 5 g/L NaCl; Difco) in 23 mL borosilicate test tubes (Thermo Fisher) and incubated at 37°C, 70% relative humidity, with rolling aeration in a TC-7 roller drum (New Brunswick Scientific). Viable cell counts were determined using the spot titering assay and are accurate to two- to threefold with a limit of detection of <1,000 CFU/mL (40). Cultures were plated onto LB agar plates with or without antibiotics, as appropriate (kanamycin 50 µg/mL; nalidixic acid 20 µg/mL; streptomycin 25 µg/mL).

### Batch culture assays

Monoculture incubations and competition assays were inoculated from overnight cultures at a dilution of 1:1,000 (vol:vol). For monoculture experiments, 5 µL of an overnight culture is inoculated into each tube; in a two-strain competition co-culture, 2.5 µL of each overnight culture is inoculated; and for a 3-strain competition tri-culture, 1.7 µL of each overnight is inoculated into 5 mL of LB. All culture growth yields and competition assays were performed at least twice independently, in triplicate, for a total of at least six replicates per experiment.

### Iron excess and depletion experiments

For conditions of iron excess, media were brought to 100 µM Fe_2_SO_4_ (Sigma) prior to inoculation. For iron depletion experiments, media were brought to 200 µM of 2,2′-dipyridyl before inoculation. The concentrations of added iron sulfate or dipyridyl were chosen after determining the minimum inhibitory concentration of each compound for the wild-type strain, ZK126 (data not shown); the highest concentration that did not affect wild-type growth was selected. All culture growth yields and competition assays were performed in triplicate, at least twice independently, for a total of at least six replicates per experiment.

### Siderophore assay

Overnight cultures were inoculated into 5 mL LB 1:500 (vol:vol) in the absence or presence of 200 µm 2,2′-dipyridyl. As each siderophore assay requires 1 mL per timepoint, eight identical cultures per strain and condition were prepared to ensure that culture volume change from sampling did not interfere with siderophore concentration measurement; therefore, each culture tube was sampled only once. One tube was sacrificed for sampling every 4 hours, over 24 hours, and pelleted in a 2 mL Eppendorf tube (VWR) at 13,000 RPM for 10 minutes. Supernatants were then assayed for siderophore production using the Chrome-Azurol S Liquid Assay (41), with the CAS shuttle solution. Arbitrary siderophore units per colony-forming unit were recorded and calculated 20 minutes after mixing all reagents (42). Viable cell counts were calculated using the spot titering assay, detailed above. Siderophore assays with the addition of 2,2′-dipyridyl were performed once in duplicate and once in triplicate for a total of 5 replicates.

### Quantitative PCR analysis

Overnight cultures of wild-type and each mutant strain were inoculated into 5 mL of LB 1:1,000 (vol:vol) and incubated at 37°C for 24 h. Five hundred microliters of each culture was mixed with 1 mL of RNA Protect (Qiagen) and incubated at room temperature for 5 minutes. The mixtures were then centrifuged at 7,500 RPM for 10 min, and pellets were resuspended in lysis buffer; 30 mM Tris-HCl, 1 mM EDTA, pH 8.0, 15 mg/mL lysozyme, 9 mAU/mL proteinase K (Qiagen). Resuspensions were incubated at 37°C for 30 minutes, and total RNA extraction of each lysate was carried out through the RNeasy Mini kit (Qiagen) using the manufacturer’s protocol. Four hundred nanograms of purified total RNA of each strain was reverse transcribed using a qPCRBIO cDNA synthesis kit (PCR Biosystems). A mix of 2 µL (20 ng) cDNA, 1 µL each of forward and reverse primers (10 µM), and 10 µL of SyGreen Mix Lo-ROX (PCR Biosystems) were analyzed on an Eppendorf Mastercycler ep Realplex. The relative fold change was calculated via the 2^−ΔΔCT^ method (43) using *ihfB* as an internal reference for data normalization.

### Statistical analysis

All survival and fitness measurements, siderophore assays, and RT-qPCR were conducted at least two times independently, with replicates. For all experiments, three liquid cultures (*n* = 3) were used per strain per condition. RT-qPCR experiments were conducted with three biological replicates and two technical replicates. For all, but the three-strain competition survival plot and RT-qPCR, an unpaired, statistical significance was calculated by two-tailed *t*-test. For the three-strain competition survival measurements, an ANOVA statistical test, and for RT-qPCR a Mann-Whitney *U* test, was performed for statistical significance.

## RESULTS

### *ftnA* and *dps* mutant strains exhibit survival deficits when incubated in monoculture

**Fig 1:**
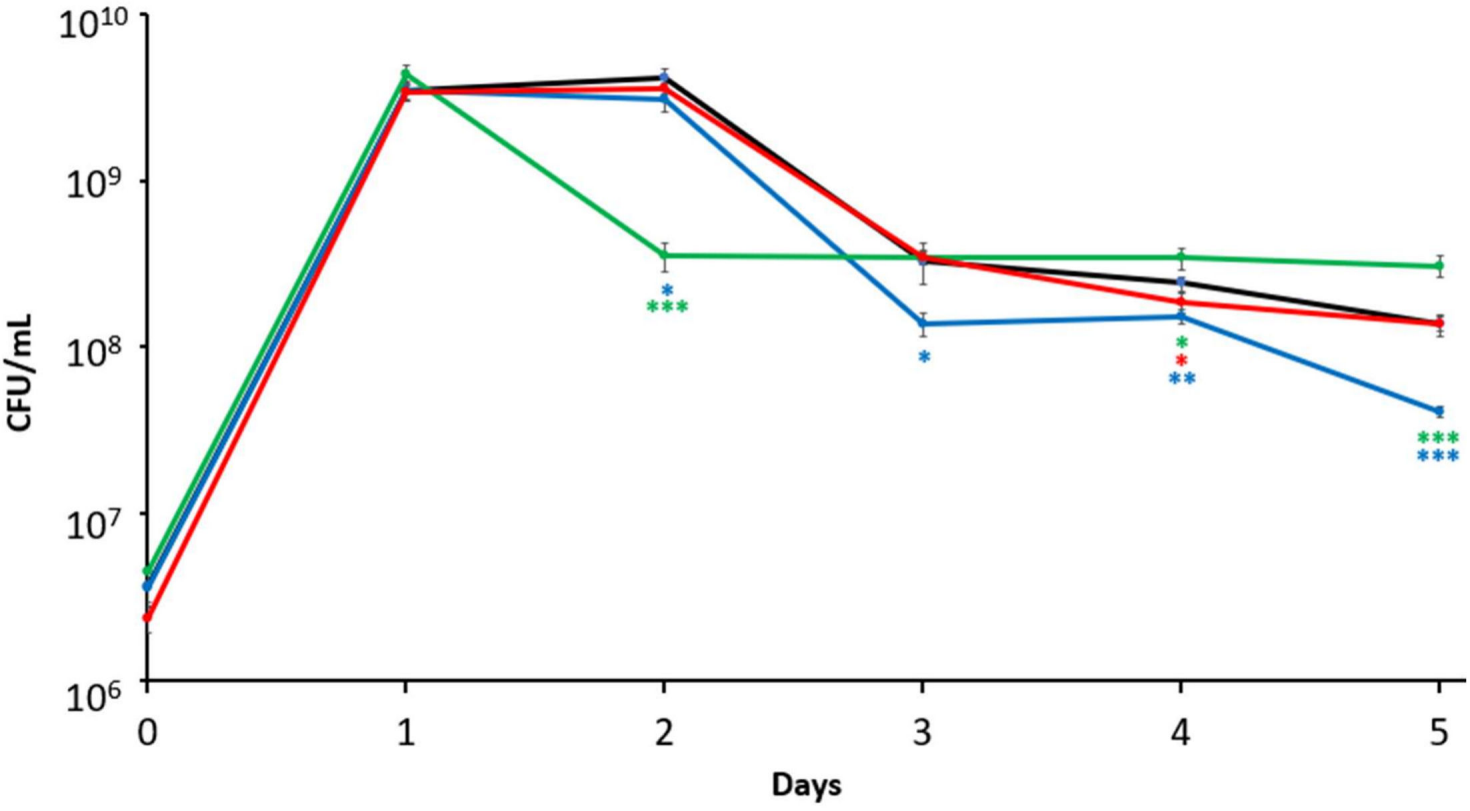
Mutations in the ferritins *ftnA* and *dps* impair survival in stationary phase and LTSP, respectively. Viable cell counts of monocultures of wild-type cells (black) and strains mutated for *ftnA* (green), *bfr* (red), or *dps* (blue) were measured over 5 days. Statistical significance (unpaired *t*-test, two-sided) is represented relative to the wild-type strain. Data are mean ± s.d.; *P < 0.05; **P < 0.05; ***P < 0.0005 (*n* = 6 cultures per strain).

To assess the relative contributions to long-term survival of each ferritin in *E. coli,* mutant strains lacking each ferritin (*ftnA, bfr,* or *dps*) were inoculated into fresh LB medium in monoculture. Viable cell counts were measured daily for 5 days, along with a wild-type control (Fig. 1). All strains have a peak population density of ~4 × 10^9^ colony-forming units per mL (CFU/mL) upon entry into stationary phase. The wild-type strain exhibits a 2-day stationary phase, with a 10-fold population decline during death phase and ultimately maintains ~2 × 10^8^ CFU/mL in LTSP. The *bfr* mutant maintains cell densities comparable to the wild-type strain, exhibiting a similar stationary phase, death phase, and LTSP survival profile. The *ftnA* mutant enters death phase 1 day earlier than the other strains, exhibiting a 10-fold loss of viability on day 2, but ultimately stabilizes at a higher population density of ~5 × 10^8^ CFU/mL in LTSP. The *dps* mutant exhibits a 2-day stationary phase with ~15-fold decrease in viable cell counts at the end of death phase on day 3. Upon entry into LTSP, the population density of the *dps* mutant was initially ~2 × 10^8^ CFU/mL on day 4; however, unlike the other three strains, viable cell counts of the *dps* mutant declined to ~4 × 10^7^ CFU/mL on day 5, showing the lowest rates of long-term survival of the four strains tested.

### *bfr* and *dps* mutant strains exhibit lower competitive fitness against the wild type during stationary phase

**Fig 2:**
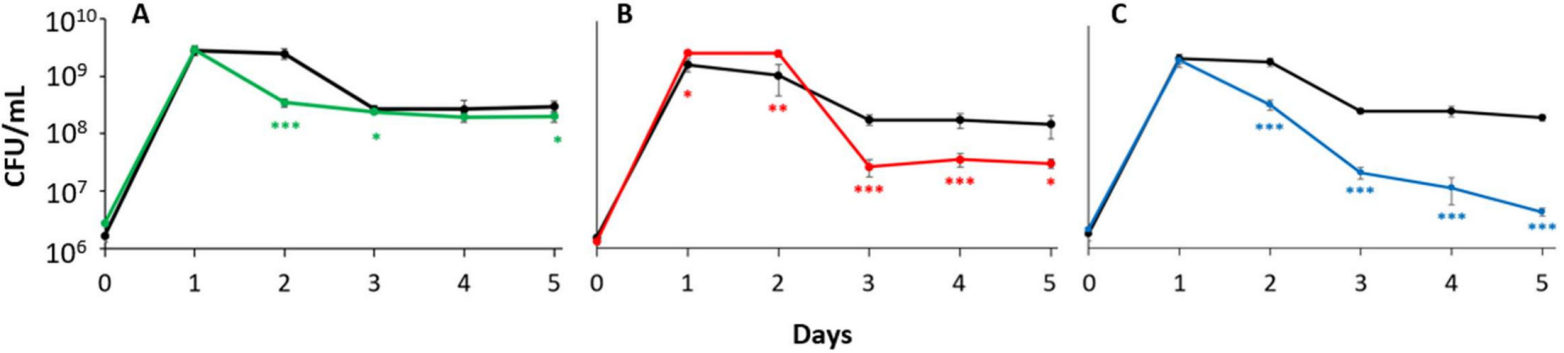
*dps and bfr* mutant strains exhibit decreased fitness during competition in stationary phase and LTSP. Viable cell counts from 5-day co-culture competition between wild type (black) and (**A**) the *ftnA* mutant strain (green), (**B**) the *bfr* mutant strain (red), or (**C**) the *dps* mutant strain (blue). Data are mean ± s.d.; *P < 0.05; **P < 0.05; ***P < 0.0005 (*n* = 6 cultures per strain).

Co-culture experiments were performed to identify any competitive growth advantages attributable to the expression of each ferritin. *ftnA, bfr,* and *dps* mutant strains were each co-inoculated with wild-type cells, and viable cell counts of each population were measured daily for 5 days. The growth and survival of the *ftnA* mutant strain was unperturbed in the presence of wild-type cells, displaying a pattern similar to that of its monoculture growth (Fig. 2A). The *bfr* mutant strain shows a reduction in competitive fitness, exhibiting a 10-fold lower population density than wild type, eventually maintaining ~2 × 10^7^ CFU/mL from death phase throughout LTSP (Fig. 2B). As previously reported (20, 21), the *dps* mutant strain displays a significant decrease in competitive fitness, with a continuous 10-fold loss in viable cells each day after a 1-day stationary phase, which was not previously observed in the monoculture, reaching ~5 × 10^6^ CFU/mL on day 5; an overall 100-fold difference from the wild-type competition (Fig. 2C).

### *dps*^−^ mutants are less fit when co-cultured with *ftnA*^−^ and *bfr*^−^ strains

**Fig 3:**
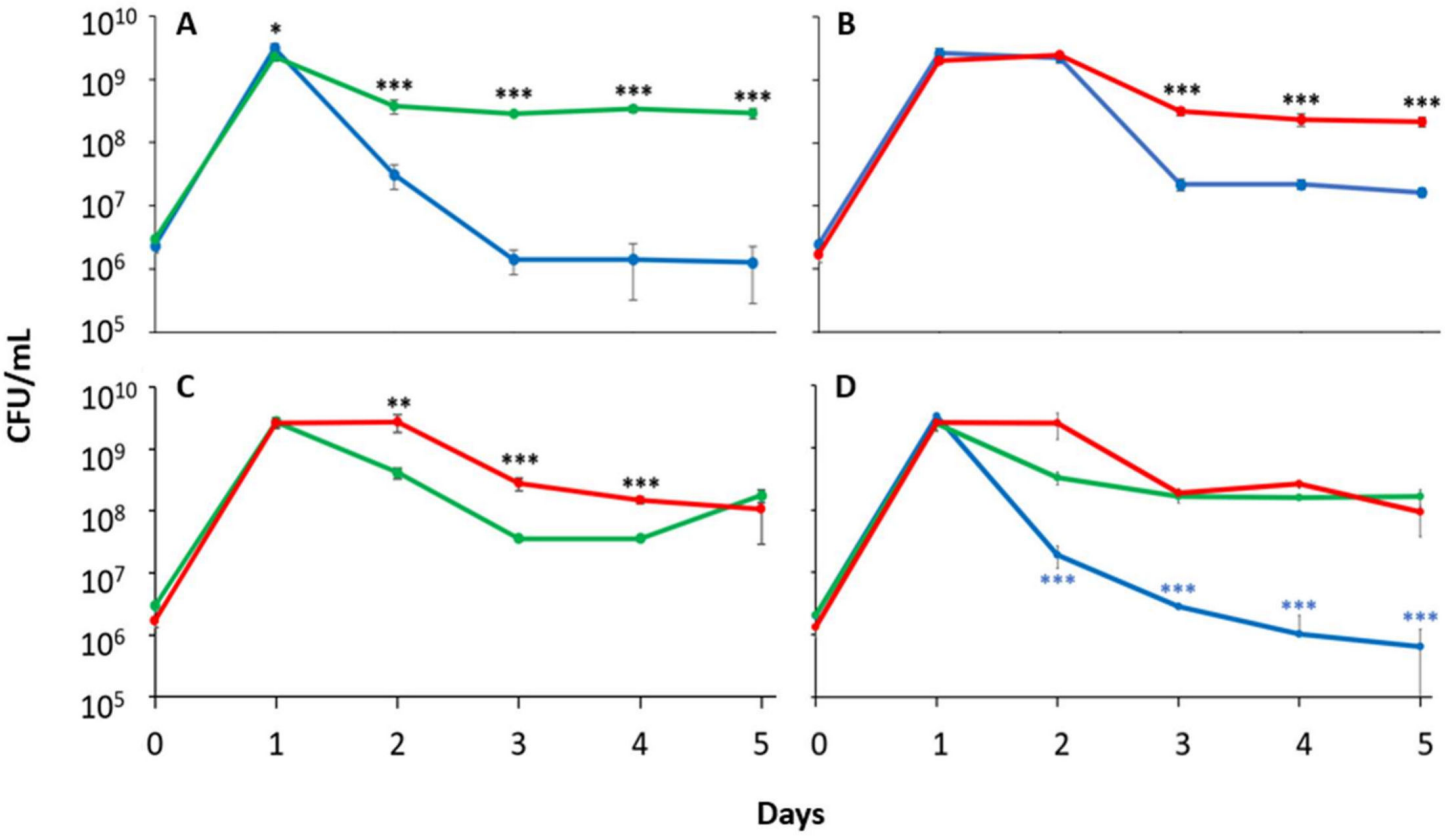
The *dps* mutant exhibits reduced competitive fitness. Viable cell counts from 5-day co-culture competition between (**A**) the *ftnA* mutant strain (green) competing with the *dps* mutant strain (blue), (**B**) the *bfr* mutant strain (red) competing with the *dps* mutant strain (blue), and (**C**) the *ftnA* mutant strain (green) competing with the *bfr* mutant strain (red). (**D**) Three-strain competition between all three ferritin mutant strains: *ftnA* (green)*, bfr* (red)*, and dps* (blue). Data are mean ± s.d.; *P < 0.05; **P < 0.05; ***P < 0.0005 (*n* = 6 cultures per strain).

Competitive coculture assays between each of the ferritin mutants were performed to elucidate any potential temporal roles for a given protein, as well as the overall relative importance of each individual ferritin under competitive conditions. The *dps* mutant strain exhibits the lowest competitive fitness among the three ferritins. When co-inoculated with the *bfr* mutant strain, the *dps* mutant exhibits a population density at least 10-fold lower than the *bfr* mutant after a 2-day stationary phase, leveling at ~1.5 × 10^7^ CFU/mL (Fig. 3B). The *bfr* mutant exhibits growth similar to that during monoculture. The loss of fitness of the *dps* mutant was further observed during competition against the *ftnA* mutant, where both strains exhibit a 1-day stationary phase. Upon the transition into LTSP, the *dps* mutant exhibits at least 100-fold lower population density than the *ftnA* mutant strain, reaching ~2 × 10^6^ CFU/mL while the *ftnA* mutant exhibits growth patterns similar to that observed in monoculture (Fig. 3A).

In a coculture of the *ftnA* and *bfr* strains, the *ftnA* mutant exhibits a 1-day stationary phase, while the *bfr* strain shows a 2-day stationary phase. The extent of the death phase initially experienced by the *ftnA* population was more severe than that of the *bfr* strain and what was observed in monoculture; however, both cultures ultimately reach the same population density of ~10^8^ CFU/mL by day 5 (Fig. 3C).

To further investigate Dps’ contributions to long-term survival, a three-strain competition assay was conducted with the ferritin mutants (Fig. 3D). The *ftnA* and *dps* strains underwent a 1-day stationary phase as opposed to the *bfr* mutant’s 2-day stationary phase. After the stationary phase, each mutant enters a death phase, with the *dps* mutant exhibiting the steepest decline in viability. Furthermore, the *dps* mutant population never recovers from the death phase and decreases to ~2 × 10^5^ CFU/mL by the end of the experiment, representing a more than 1,000-fold difference from its peak density of ~2 × 10^9^ CFU/mL, while the *ftnA* and *bfr* strains maintain a population density of ~3 × 10^8^ CFU/mL in LTSP, similar to that observed under monoculture conditions.

### *dps* mutants show increased sensitivity to excess iron compared to *bfr* or *ftnA* mutant strains

**Fig 4:**
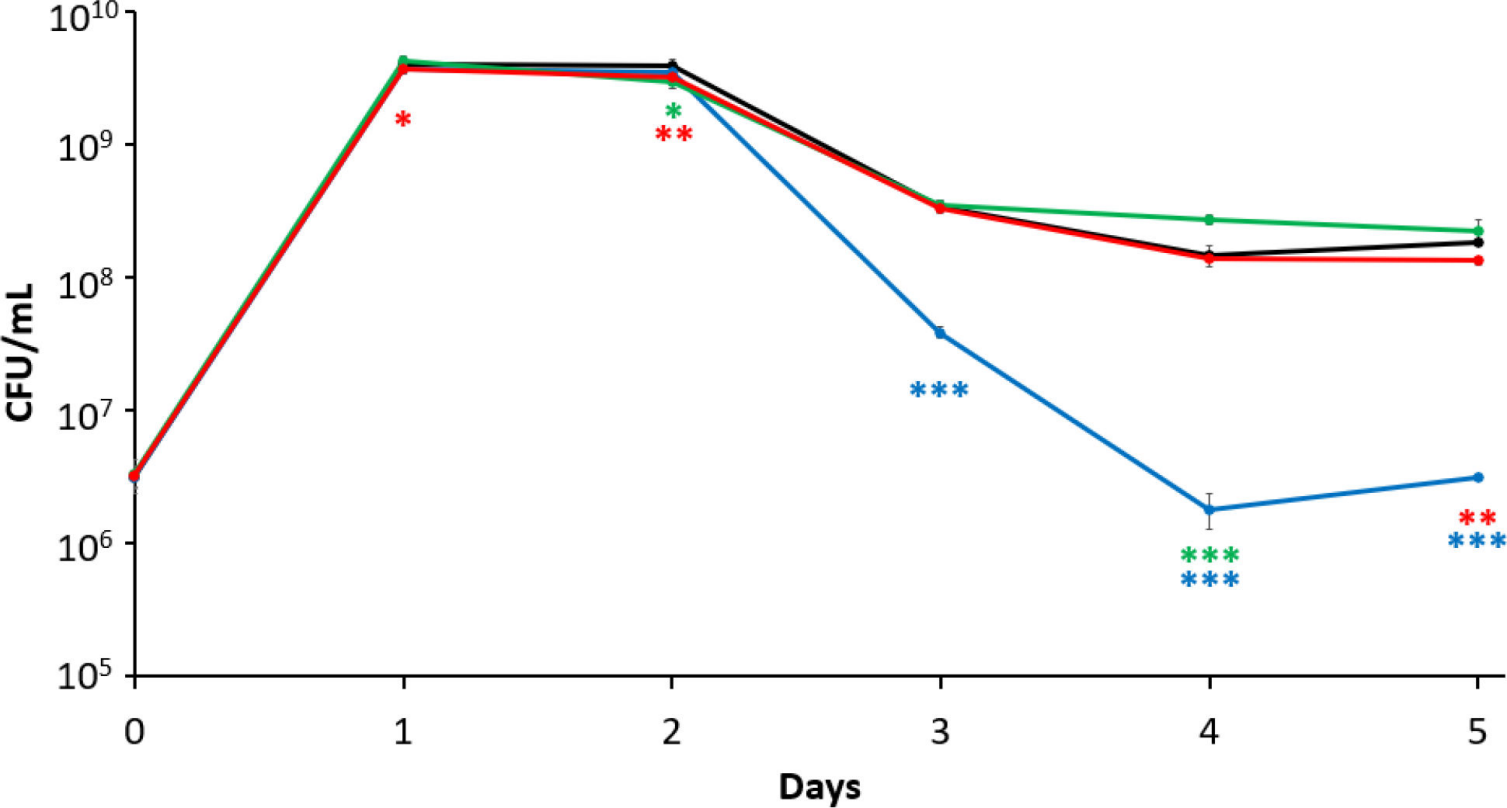
Loss of Dps increases sensitivity to iron toxicity and decreases survival during LTSP. Viable cell counts of monocultures supplemented with 100 µm Fe_2_SO_4_ of wild-type cells (black) and strains lacking *ftnA* (green), *bfr* (red), or *dps* (blue) were measured for 5 days. Statistical significance (unpaired *t*-test, two-sided) is represented relative to the wild-type strain. Data are mean ± s.d.; *P < 0.05; **P < 0.05; ***P < 0.0005 (*n* = 6 cultures per).

To assess the iron detoxification functionality and relative role for each ferritin under conditions of excess iron, monocultures were prepared in the presence of 100 µM Fe_2_SO_4_ and viable cell counts were determined daily for 5 days (Fig. 4). The *ftnA* and *bfr* mutant strains exhibit growth and survival patterns similar to the wild type, with the exception of the *ftnA* mutant displaying a 2-day stationary phase, unlike the 1-day stationary phase found in monoculture or competition assays under standard conditions. All strains reach a peak population density of ~2 × 10^9^ CFU/mL with a subsequent 10-fold decline during the death phase. The wild-type, *ftnA,* and *bfr* strains then maintain a population density of ~2 × 10^8^ CFU/mL throughout LTSP. In contrast, while maintaining a 2-day stationary phase, the *dps* mutant strain experiences more than 10-fold greater loss of viability during death phase compared to its growth in monoculture under standard conditions. This substantial decline continues until day 4, where the population density levels off at ~3 × 10^6^ CFU/mL, indicating that the *dps* mutant is 100-times more sensitive to excess iron than the other ferritin mutants.

### *dps* shows greater sensitivity and greater siderophore production in response to iron starvation

**Fig 5:**
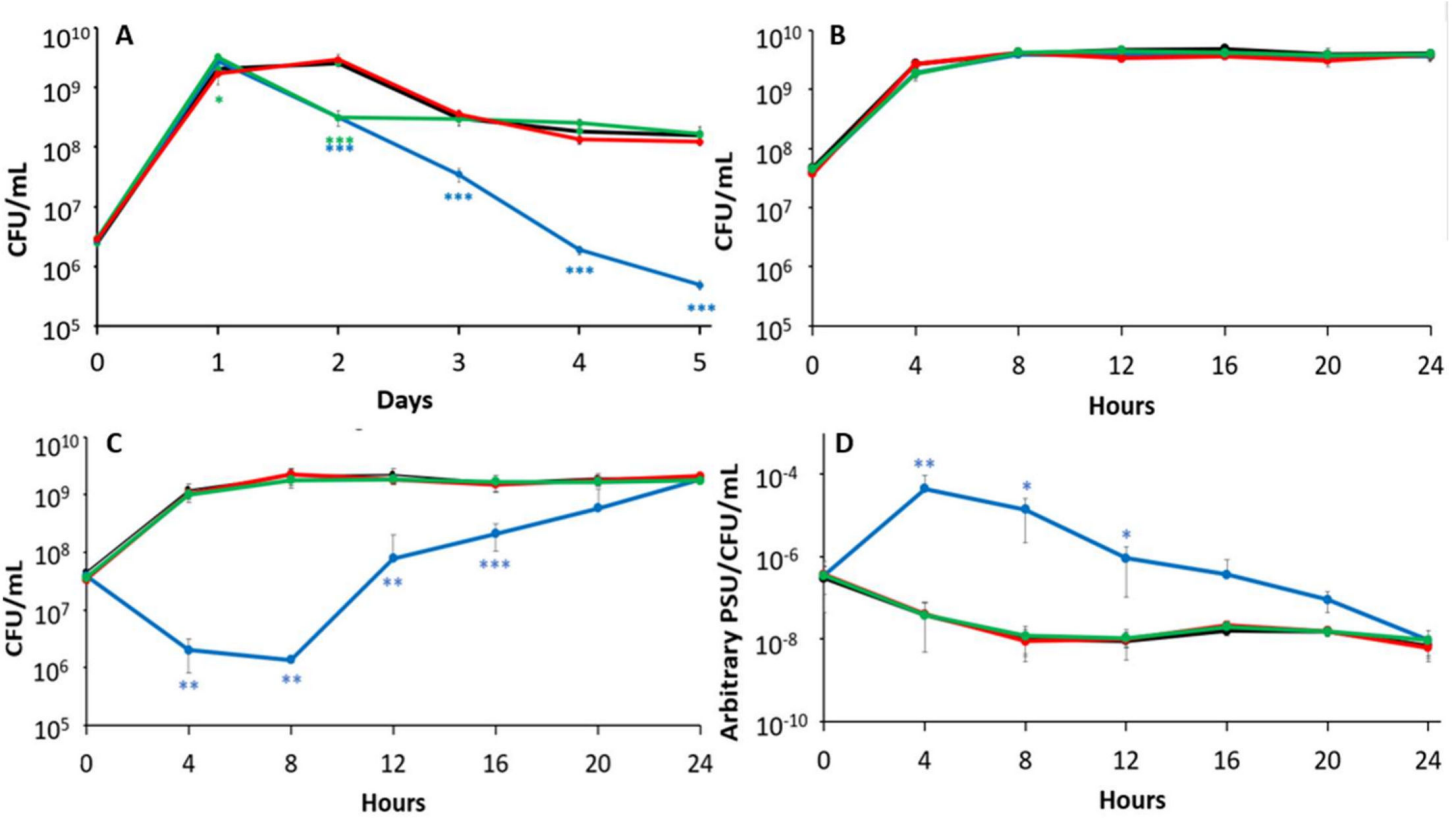
The *dps* mutant strain exhibits sensitivity to iron starvation and increases siderophore production. Viable cell counts of monocultures (**A**) supplemented with 200 µM 2,2′-dipyridil of wild-type cells (black) and strains lacking *ftnA* (green), *bfr* (red), or *dps* (blue). (**B**) 24 hour outgrowth in monoculture of wild-type cells (black) and strains lacking either *ftnA* (green), *bfr* (red), or *dps* (blue). (**C**) 24 hour outgrowth of monocultures supplemented with 200 µM 2,2′-dipyridil of wild-type cells (black) and strains lacking either *ftnA* (green), *bfr* (red), or *dps* (blue). (**D**) Time course of siderophore production in monocultures inoculated into medium supplemented with 200 µM 2,2′-dipyridil. Cultures were sampled every 4 hours for 24 hours. Statistical significance (unpaired *t*-test, two-sided) is represented relative to the wild-type strain. Data are mean ± s.d.; *P < 0.05; **P < 0.05; ***P < 0.0005 (*n* = 5 cultures per strain).

To assess the contribution of each ferritin to help maintain cellular iron metabolism via iron reservoir function, each mutant was inoculated into media containing the iron chelator 2,2′-dipyridyl, to induce iron starvation (43, 44) and viable cell counts were determined daily for 5 days. The *ftnA* mutant exhibits a similar growth pattern to its monoculture in standard LB, with a 1-day stationary phase, a death phase with a 10-fold population decline, and an overall higher population density (5 × 10^8^ CFU/mL) relative to the wild type through LTSP. The *bfr* mutant is similar to the wild type, experiencing a 2-day stationary phase, with a 10-fold decline in viability upon the transition into death phase, and maintaining a population density ~2 × 10^8^ CFU/mL throughout LTSP. In contrast, the *dps* mutant exhibits a significant survival deficit in the presence of the iron chelator, entering death phase after Day 1 instead of Day 2, and showing population decreases four orders of magnitude larger than the other strains, declining to ~2 × 10^5^ CFU/mL during LTSP (Fig. 5A).

To further investigate the role of each protein in iron storage, the outgrowth of wild-type and ferritin mutant strains was assessed immediately after inoculation into standard or iron-chelated LB and viable cell counts were determined every 4 hours over a 24 hour period. In standard LB, no differences in growth patterns were observed (Fig. 5B). However, in iron-chelated LB, while all strains eventually reach ~5 × 10^9^ CFU/mL after 24 hours, peak population density is observed within 4 hours post inoculation for all strains except the *dps* mutant, which exhibits growth yields 100-fold lower at that time. This difference increases to ~1,000-fold by 8 hours after inoculation (Fig. 5C).

Since the production of siderophores has been linked to the cell’s perception of its iron status, it can serve as a proxy to gauge if a cell’s iron requirements are being satisfied (27, 29, 44, 45). During outgrowth, siderophore concentration in wild-type and ferritin mutant cultures were determined for cells grown in either standard or iron-chelated LB. Immediately after inoculation, siderophore concentrations for all strains are similar in both media. In standard LB, there is an ~100-fold decline of siderophore levels over an 8 hour period, gradually increasing back to the initially observed levels by 24 hours. Under iron-starvation conditions, the *dps* mutant exhibits a more than 2,000-fold increase in siderophore production compared to the wild type or either canonical ferritin mutant strain through 8 hours post inoculation. The siderophore concentration of the *dps* mutant slowly returns to similar levels as the other strains by 24 hours (Fig. 5D).

### Dps plays a compensatory role under different conditions of iron availability in *ftnA* and *bfr* mutants

**Fig 6:**
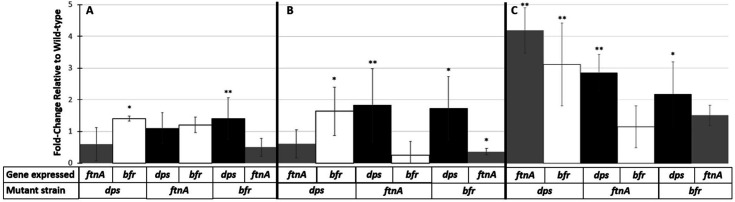
Ferritin gene transcription in mutant strains. qPCR analysis of 24 hour cultures with fold change relative to wild type. *dps* (black), *ftnA* (gray), and *bfr* (white) cultures were grown (**A**) under standard conditions, (**B**) supplemented with 100 µm of Fe_2_SO_4_, or (**C**) supplemented with 200 µm of 2,2′-dipyridil. Statistical significance (Mann-Whitney *U* test) is represented relative to the wild-type strain. Data are mean ± s.d.; *P < 0.05; **P < 0.05; ***P < 0.0005 (*n* = 3 cultures per strain).

To further explore the significant role of Dps under conditions of iron excess and starvation, as well as elucidate potential compensatory roles for each ferritin, transcript levels were determined using qPCR on the wild-type and ferritin mutant strains after 1-day of incubation under different conditions of iron availability. The expression of each ferritin was compared to their respective expression in the wild type when incubated under the same conditions. When incubating under standard conditions, the *dps* mutant shows an ~50% upregulation of *bfr* transcripts while *ftnA* transcription was downregulated by 45%–95%. In the *ftnA* mutant, the number of *dps* and *bfr* transcripts shows an increase of 20%–50%, while in the *bfr* mutant, the number of *dps* transcripts is more than twice that in the wild type, and *ftnA* is downregulated 20%–80% (Fig. 6A).

Under conditions of iron toxicity, the *dps* mutant shows a 50%–100% upregulation of *bfr* and 45%–80% downregulation of *ftnA*. Both the *ftnA* and *bfr* mutants show an almost twofold increase in *dps* expression with a decrease of the other respective ferritin (Fig. 6B). Under conditions of iron starvation, in the *dps* mutant, the transcript levels of *ftnA* and *bfr* increase four- and threefold, respectively. In the *ftnA* mutant, *dps* expression is almost threefold greater, while *bfr* is unaffected. In the *bfr* mutant*, dps* and *ftnA* expression increases, with *dps* transcripts increasing ~2-fold compared to wild type (Fig. 6C).

## DISCUSSION

*E. coli* expresses three genes that encode proteins with ferritin-like activity (*ftnA, bfr,* and *dps*) that serve the common functions of iron storage and iron detoxification to manage Fe(II) availability and the production of reactive oxygen species. Here, we elucidate the roles for each of the three proteins during all phases of the bacterial lifecycle in the laboratory and, specifically, the importance of Dps as a ferritin homolog by investigating its role in long-term survival. While each ferritin contributes to growth advantage, competitive fitness, and iron metabolism, data presented here support a model where Dps serves as the crucial ferritin-like protein, essential for mitigating stresses resulting from the absence of either of the two canonical ferritins.

In monoculture, the *ftnA* mutant strain experiences a stationary phase lasting only 1 day unlike the other mutants that exhibit the 2-day long stationary phase typical of this parental strain. These data point toward temporal differences could exist in the expression patterns and relative importance of each ferritin. FtnA is expressed in early stationary phase (46) and removing its ferritin-activity results in observable differences at that phase. However, while also expressed in stationary phase, the *dps* mutant’s survival defect is not observed until after death phase and entry into LTSP, while the *bfr* mutant shows no observable defect. This may indicate each ferritin’s importance for survival during distinctive phases, potentially providing different levels of growth advantage depending on the needs of the cell at that time. It is important to note that the observed growth phenotypes of the ferritin mutants are not due to a buildup of toxic metabolites (Fig. S1), nor should be attributed to changes in pH, as all cultures of all strains had identical pH values across all conditions tested (data not shown). Other differences in batch culture survival, supporting this model, become apparent after stationary phase. The *ftnA* and *bfr* mutant strains appear relatively unaffected by the loss of their respective ferritin-activities, while the *dps* mutant struggles to maintain similar population densities during long-term stationary phase. This survival pattern for the ferritin mutants incubated in monoculture is also observed in the LTSP population density of the double and triple mutants. If a strain lacks *dps,* it struggles to reach high population densities compared to the strains still encoding *dps* (Fig. S2).

Competition assays were employed to evaluate the importance of each ferritin during each growth phase. The *ftnA* strain exhibits a 1-day stationary phase while in competition with the wild type, but maintains comparable population densities throughout LTSP, further suggesting a potential role early in stationary phase. However, after death phase, FtnA appears to become more dispensable. The *bfr* strain shows an opposite temporal advantage, with equivalent population densities to the wild type during stationary phase, but a loss of competitive fitness after death phase. This may indicate that this ferritin’s role is more important during late stationary phase and LTSP. Co-culture of the *ftnA* and *bfr* strains highlights the proposed temporal hierarchy, with the *bfr* strain showing higher competitive fitness initially, which then declines during the transition into LTSP, when the *ftnA* strain reaches similar cell densities. The *dps* mutant, in comparison, exhibits the lowest competitive fitness and viability as lower population densities are observed when co-inoculated with the wild-type, *ftnA,* or *bfr* strains, and a 1-day stationary phase when *dps* mutants are co-inoculated with the wild-type or *ftnA* strains. This loss of competitive fitness becomes particularly clear in the triculture competition, suggesting the potential essentiality of Dps in natural environments. Furthermore, monoculture growth patterns and competitive growth advantages are consistent with observed transcriptomic patterns for the three ferritins in a K-12 *E. coli* strain. Expression of *ftnA* is the highest in late log phase, dropping significantly upon entry into stationary phase, while the highest expression of *bfr* and *dps* begins at early stationary phase and continues into LTSP, with *dps* having a substantially larger number of transcripts at any given time (47). This parallel between transcript levels and observed mutant strain growth patterns further supports a model where there is a temporal role for each ferritin, coupled with the importance of Dps’ ferritin-activity in mitigating stress, especially in the absence of the other canonical ferritins.

Given that the *dps* mutant exhibits significant survival and competition defects under standard culture conditions, we performed experiments to understand the role of each ferritin during the stresses of iron excess or depletion. We hypothesize that the Dps dodecamer may be the most fundamental factor in maintaining iron homeostasis, potentially modulating the bulk of cellular iron metabolism under both extremes of iron availability. This may be due both to Dps being more abundant than the other two ferritins from stationary phase through LTSP and to that fact that Dps primarily uses H_2_O_2_ as the oxidant for its ferroxidase activity. In support of this model, under conditions of high iron concentration, the wild-type and *bfr* mutant strain are essentially unperturbed and show growth and survival patterns similar to that of monocultures in standard LB. The *ftnA* mutant strain also exhibits similar survival and growth patterns, with the exception of now displaying a 2-day-long stationary phase. We speculate that this additional day in stationary phase may be due to the induction of *dps* expression via the OxyR response to elevated oxidative stress (48). This OxyR response subsequently produces an abundance of Dps, which would mitigate the toxic effects of both H_2_O_2_ and Fe(II) concentrations in the cell, potentially compensating for the lack of ferritin activity from the *ftnA* mutant in early stationary phase. The *dps* mutant exhibits the largest population decline of all the strains further reinforcing its significance as a potential iron sink.

The results of the qPCR analysis provide further insight into the potential compensatory function of Dps and the ferritin proteins under varying conditions of iron availability. Under standard conditions, the *dps* mutant displayed a marginal upregulation of *bfr* transcripts, accompanied by a downregulation of *ftnA* transcript*,* suggesting a phase-appropriate response to the loss of a Dps, as Dps and Bfr are primarily expressed during late stationary phase in wild-type cells. The *ftnA* mutant showed slight increases in both *dps* and *bfr* transcripts, while the *bfr* mutant exhibited a significant twofold upregulation of *dps* synthesis, coupled with a downregulation of *ftnA*. This could indicate that the loss of one of the other ferritins may trigger the upregulation of Dps as a compensatory mechanism. Under conditions of iron toxicity, the *dps* mutant showed subtle changes, with a slight increase in the amount of *bfr* transcripts and a decrease of *ftnA*, similar to the expression profile of the *dps* mutant incubated in standard LB. In contrast, both the *ftnA* and *bfr* mutants demonstrated significant increases in *dps* transcription, accompanied by decreased levels of the other respective ferritins, further suggesting that Dps may play a compensatory role under stress conditions. Notably, during iron starvation, *ftnA* and *bfr* expression significantly increased, with *ftnA* showing a fourfold and *bfr* a threefold increase of transcripts in the *dps* mutant. This supports a model where Dps plays a crucial role as an iron reservoir, as it is hypothesized that without Dps, *E. coli* will overexpress the other two canonical ferritins, apparently to replenish potential iron storage ability. In the *ftnA* mutant, *dps* transcripts increased nearly threefold, whereas *bfr* transcripts remained unchanged. Meanwhile, in the *bfr* mutant, both *dps* and *ftnA* transcripts were elevated, with *dps* showing a twofold increase compared to wild type. The data suggest that Dps may serve as a key regulator in maintaining iron homeostasis in the absence or dysfunction of the ferritin proteins, particularly when iron is limited or in excess.

When introduced into a culture environment with decreased extracellular iron availability, it is hypothesized that Bfr and FtnA release their reservoir of Fe(III) as a compensatory response (49). To begin to elucidate the individual contributions of each ferritin as a potential iron storage molecule and subsequent source of intracellular iron, cells were incubated under iron-starvation conditions. All strains, except the *dps* mutant, exhibit growth patterns essentially similar to those observed when cultured in standard LB. In contrast, the *dps* mutant strain has a 1-day stationary phase, previously observed only under competitive conditions, followed by the most precipitous population decline during death phase of all growth conditions. Subsequently, the *dps* mutant undergoes a continuous population decline throughout LTSP reaching the lowest LTSP population density recorded in this study, at 1,000-times less than the other strains. Notably, complementation of the *dps* mutant with a plasmid expressing *dps* under both its native promoter (18) and inducible promoter (8) partially or fully restored long-term survival to wild-type levels across all tested iron conditions (Fig. S3). Significant differences in LTSP survival patterns, with no observed variation of peak population densities, prompted further investigation of the outgrowth of each strain for the first 24 hours after inoculation. Under standard LB conditions, no variation between the strains was observed. However, under iron-starvation conditions, a substantial difference in the *dps* strain becomes evident. While the population of all strains reached similar cell density by 24 hours, the *dps* mutant initially undergoes a substantial population decline. This decline early after inoculation reinforces Dps’ role as a crucial ferritin and, specifically, its role as an iron reservoir during the transition from stationary phase back into exponential phase growth. While the average *E. coli* bacterium is estimated to contain ~10^5^ to 10^6^ iron atoms (28, 50), which could theoretically be contained in as few as ~250 of either of the canonical 24mer ferritins, based on the data presented here, we postulate that Dps plays a driving role of maintaining and regulating iron levels in environments of both iron excess or starvation extremes. As the most abundant of the three ferritins, Dps has the capacity to sequester and store large, potentially toxic quantities of iron, allowing the cell to mitigate the deleterious effects of iron excess, and release it when cells are under iron starvation stress or require higher intracellular iron concentrations due to the transition into log phase. Therefore, we hypothesize that the absence of Dps imposes a significant pressure on cells when inoculated into an environment with reduced iron availability due to the lack of a significant intracellular iron reserve. This model is reinforced when examining siderophore production over the same 24 hour period. Under standard conditions of iron availability, each strain produces comparable concentrations of siderophore. However, in an environment lacking iron, while the *ftnA* and *bfr* mutant strains produced 5- to 20-fold greater concentrations of siderophores in comparison to the wild type, the *dps* mutant strain produced more than 2,000-fold larger concentrations of siderophore. This suggests a substantial imbalance of iron homeostasis, and potentially the cell’s overall inability to maintain iron metabolism during iron starvation without Dps present. Furthermore, given the ability of Dps to bind DNA and organize the chromosome during stationary phase (18, 19), it is formally possible that some of the phenotypes attributed to Dps could be modulated through its DNA-binding activity.

This study correlates the potential relationship between iron metabolism and long-term survival in *E. coli* by elucidating the temporal role and essentiality of each ferritin, specifically the ferritin-like protein, Dps. The universal conservation of Dps among virtually all bacteria (17) sheds light on its potential as a critical protein for long-term survival in a variety of environments. While being dual-functioned in many species, the data presented here support a model where Dps ferritin activity plays a key role in cellular survival across many stressful conditions, but especially under those where ferritin-activity is potentially vital. The model we present may also provide further insights into how cells utilize Dps ferritin activity and its contribution to short- and long-term survival. Understanding these roles may influence protocols to restore or maintain human microbiota or, conversely, help in combating bacterial pathogens since many pathogens rely on the ability to utilize host and microbial iron sources during infection (51).

## SUPPLEMENTAL MATERIAL

Supplemental material spectrum.01837-24-s0001.docx Fig. S1 to S3.

ASM does not own the copyrights to Supplemental Material that may be linked to, or accessed through, an article. The authors have granted ASM a non-exclusive, world-wide license to publish the Supplemental Material files. Please contact the corresponding author directly for reuse.
