# Supplementary material for: A role for Dps ferritin activity in long-term survival of Escherichia coli
Source: Microbiol Spectr. 2025 Sep 2;13(10):e01837-24. doi: 10.1128/spectrum.01837-24 (PMC12502760; doi:10.1128/spectrum.01837-24)
Supplement: Supplemental material — Fig. S1 to S3. [file spectrum.01837-24-s0001.docx]

**SUPPLEMENTAL MATERIALS:**

**MATERIALS AND METHODS**

**Conditioned media experiments**

Overnight cultures of wild-type, *dps, ftnA,* or *bfr* strains were inoculated into five independent 5 mL tubes of LB broth. After 24 hours of incubation, cultures were pelleted, and the supernatants were passed through a washed 0.2-µM filter. Washing was done with deionized water to remove any potential metabolizable proprietary wetting agents (unpublished observation). The pH of spent media was adjusted to pH 7.4 via addition of 5M HCl. Fresh overnights of each strain were inoculated into spent media at 1:1,000,000 (vol/vol), and viable cell counts were determined daily.

**Complementation experiments**

*dps* mutant strains were complemented in two ways, using plasmid pJE106 [18] which contains the entire *dps* gene under its own promoter in pBR322 or using a pBAD18-dps plasmid [8] that overexpresses the *dps* gene upon addition of 0.1% L-arabinose. Vector-only controls utilize pBR322 or pBAD, as appropriate.

**FIGURE LEGENDS**


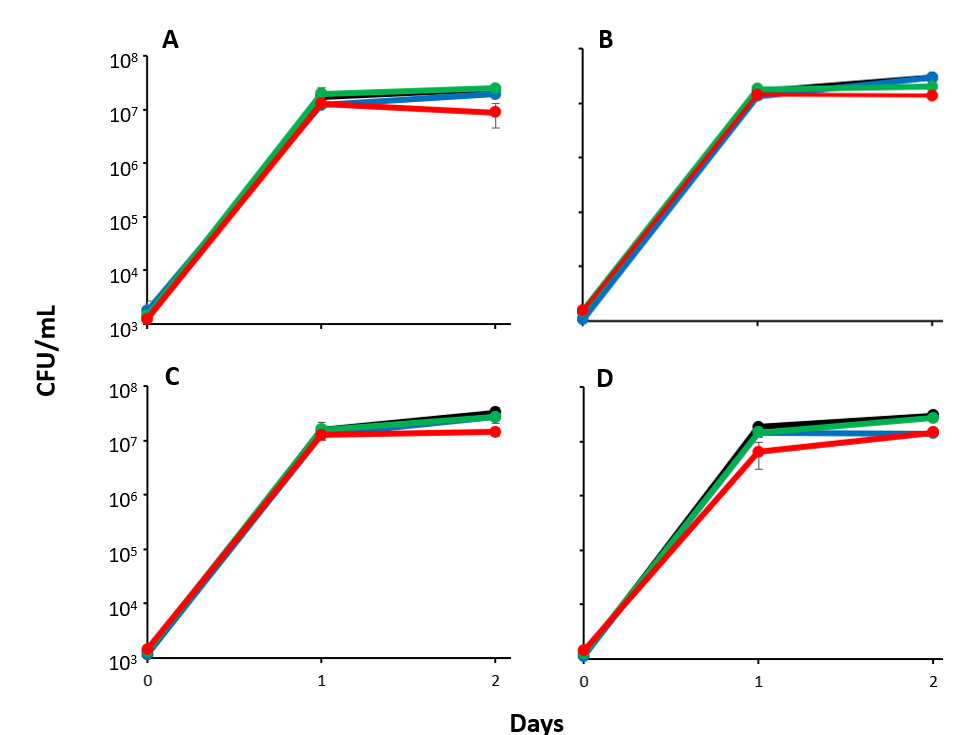


**Supplemental Figure 1: Cell growth in conditioned media.** Viable cell counts of monocultures inoculated into conditioned media. Wild-type cells (black) and strains lacking either *ftnA (*green), *bfr* (red), or *dps* (blue) were incubated for 48 hrs in (A) Wild-type conditioned medium, (B) *dps* conditioned medium, (C) *ftnA* conditioned medium, or (D) *bfr* conditioned medium. Statistical significance (unpaired *t*-test, two-sided) is represented relative to the wild-type strain. Data are mean ± s.d.; *, *P* < 0.05; **, *P* < 0.05; *** *P<0.0005* (*n* = 6 cultures per strain).


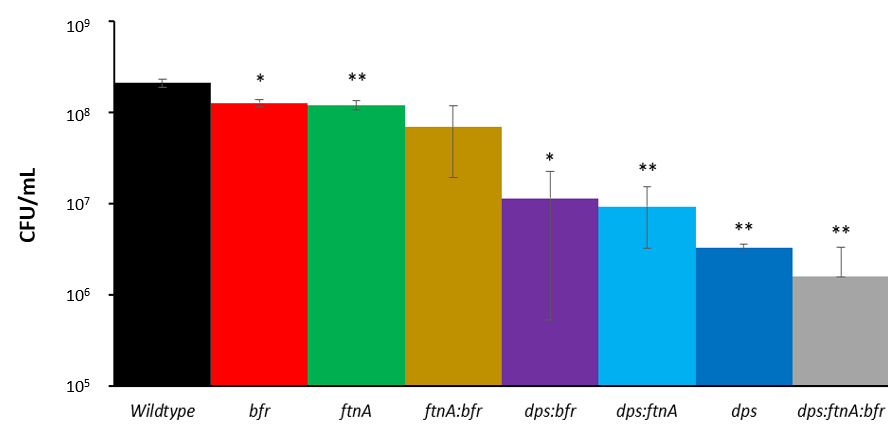


**Supplemental Figure 2: Day 5 population densities of single, double, and triple mutant strains.** Viable cell counts determined on day 5 of LTSP cultures of the following strains: wild-type cells (black), *bfr* (red), *ftnA* (green), *ftnA:bfr* (gold), *dps:bfr* (purple), *dps:ftnA*  (cyan), *dps* (blue), and *dps:bfr:ftnA* (grey). Statistical significance (unpaired *t*-test, two-sided) is represented relative to the wild-type strain. Data are mean ± s.d.; *, *P* < 0.05; **, *P* < 0.05; *** *P<0.0005* (*n* = 6 cultures per strain).


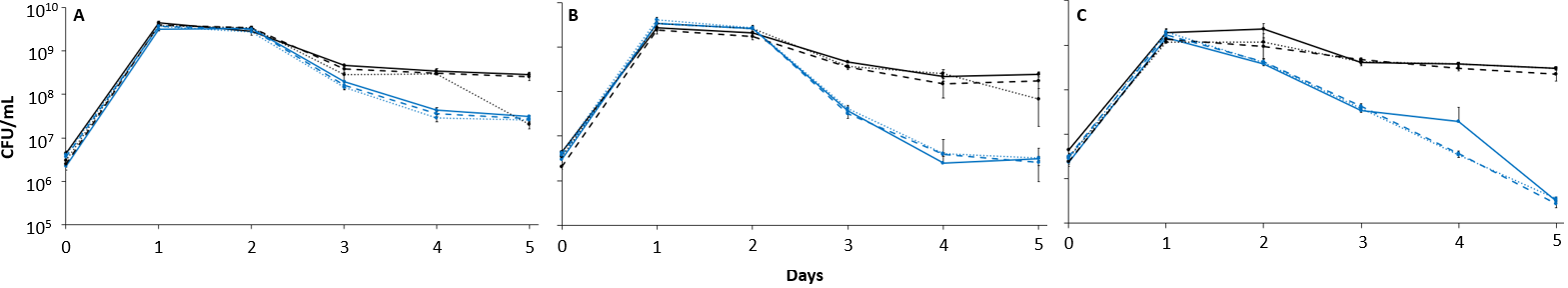


**Supplemental Figure 3: Complementation of *dps* mutants.** Viable cell counts of monocultures of wild-type (solid black), *dps* (solid blue), *dps* + pJE106 (black dotted), *dps* + pBAD18-dps with 0.1% arabinose induction at inoculation (black dashed), *dps* with pBR322 vector control (blue dotted), and *dps* with pBAD with 0.1% arabinose induction at inoculation vector control (blue dashed) were measured over 5 days under conditions of standard growth (A), excess iron (B), and iron starvation (C). Data are mean ± s.d.
